# Comparison of virtual clinical scenario and role play in learning oral pathology among dental students

**DOI:** 10.1371/journal.pone.0306712

**Published:** 2024-07-08

**Authors:** Sofia Ali Syed, Mahrukh Sheikh, Faryal Ali Syed, Saira Atif, Asifa Iqbal, Ghazia Zeeshan

**Affiliations:** 1 Department of Oral Pathology, Dr. Ishrat-ul-Ebad Khan Institute of Oral Health Sciences, Dow University of Health Sciences, Karachi, Pakistan; 2 Department of Orthodontics, Baqai Dental College, Baqai Medical University, Karachi, Pakistan; 3 Department of Oral Biology, Combined Military Hospital Lahore Medical College & Institute of Dentistry, National University of Medical Sciences, Lahore, Pakistan; 4 Department of Oral Pathology, Avicenna Dental College, University of Health Sciences, Lahore, Pakistan; King Faisal University, SAUDI ARABIA

## Abstract

In oral pathology, virtual clinical scenario illustrating dentist-patient interactions can be utilized by both students and health professionals to deliver/gain knowledge and make clinical diagnosis of oral lesions. Role play is also an educational technique which is designed to engage and motivate students in classrooms. This study aimed to compare usefulness of virtual clinical scenario and role play in learning oral pathology among second-year dental students. The students were randomly divided to one of the two groups: virtual clinical scenario group (n = 50) and role play group (n = 50). Virtual clinical scenario group was provided with virtual clinical cases of oral lesions through Google Forms whereas role play group was exposed to virtual clinical cases of oral lesions through role playing activity. Both groups underwent assessments before and after the intervention. Students’ perceptions on usefulness of both techniques in terms of diagnosis, visual parameters and impact on learning were evaluated by feedback questionnaire. Data were analyzed using Statistical Package for the Social Sciences version 27.0. Wilcoxon signed-rank test was used to compare pre-test and post-test scores. Additionally, the scores and students’ responses from both groups were compared using the Mann-Whitney U test. A P-value of < 0.05 was set as statistically significant. Students in both groups showed significantly higher post-test scores compared to their pre-test scores (P < 0.001). However, the role play group outperformed the virtual clinical scenario group, with a significantly higher post-test score (P = 0.04). Furthermore, feedback concerning role play was significantly higher than that for the virtual clinical scenario across multiple aspects (P < 0.05). Our findings suggest that role play emerges as the preferred method, significantly enhancing dental students’ learning experiences in oral pathology.

## Introduction

Traditional teaching methods including lectures, textbooks and hands-on demonstration remain indispensable in dental education. These methods enable students to understand core concepts of the subject and develop practical skills, while also facilitating direct interaction with teachers for clarification of complex topics. However, they tend to be teacher-centered, promote passive learning and provide limited real-time feedback [1].

In recent years, there has been a paradigm shift from traditional teaching towards digital education. The onset of COVID-19 pandemic followed by the closure of all educational institutions can be recognized as the primary catalyst that led to the introduction of digital education [2]. Digital education or e-learning is a teaching method that utilizes electronic media and information technology to support and enhance learning. It can be delivered through various platforms including web-based courses, mobile applications and virtual classrooms [3]. It has several potential advantages such as flexibility, ease of access along with adequate interaction and it can be cost effective for both parents and students [4].

While the era of COVID-19 pandemic is long over, digital education still remains a preferred teaching modality for many students as it effectively imparts knowledge while sustaining individual interest. This is particularly evident in the field of dentistry, where innovative digital technologies are reshaping both teaching methods and clinical practice. These digital technologies, which utilize artificial intelligence for image processing and analysis, are primarily designed to the needs of dental education. Not only do they facilitate active learning through interactive modules and virtual simulations, but they also serve practical purposes, such as providing presumptive diagnoses of various oral conditions. This integration of digital technology into dental education and practice highlights its key role in enhancing student learning and preparing them for real-world clinical settings [5–7].

Oral Pathology is an integral basic (non-clinical) science subject. In our institute, it is taught during the second-year of preclinical phase in the Bachelor of Dental Surgery (BDS) program and therefore does not involve direct interaction with patients or outpatient department. Without clinical rotation/patient interaction, students express least interest compared to clinical subjects.

Furthermore, lectures, tutorials and text-book based learning—the traditional way of teaching oral pathology, makes this subject extremely volatile. Various challenges are encountered by dental students in learning oral pathology including lack of interactive teaching methods, difficulties in diagnostic skills, lack of clinical correlation, as well as limited resources amongst others [8]. While traditional teaching cannot be replaced, e-learning holds the potential to revolutionize the entire field of dental education. Integrating digital or interactive method not only fosters student motivation but also provides meaningful educational experience in theoretical and clinical domains. Furthermore, utilizing e-learning for continuing dental education, assessments, case description as well as treatment strategies, offers an effective avenue for raising the standards of dental education [9].

A growing body of literature demonstrates that virtual learning is more effective in dental education than traditional lectures in enhancing student performance and satisfaction, underscoring the need for a blended approach that incorporates both face-face instruction and online activities [10–13].

Virtual interactive education in classrooms, demonstrating dentist-patient interactive clinical scenarios, has emerged as a highly effective teaching method that has gained widespread popularity. This method encompasses a variety of techniques designed to simulate real clinical situations, helping students develop their diagnostic capabilities and communication skills [14]. It represents a significant advancement from traditional clinical or virtual scenario visualization, as it exposes students to the environment of an actual clinic in a more realistic manner. Furthermore, these virtual clinical scenarios can be utilized by students and dental professionals for knowledge retention and diagnosis of oral lesions, respectively. Similarly, role play is an educational technique designed to actively engage and motivate students in the classrooms. For example, in this approach, students assume the role of dentists, while teachers act as patients, thereby facilitating interactive teaching [1, 15, 16].

In Pakistan, prior research has indicated improved student performance in oral pathology with problem-based learning and blended learning approaches [17, 18]. However, there remains a dearth of studies exploring the effectiveness of role play activities and virtual clinical scenarios within the context of oral pathology education. These innovative teaching methods have the potential to enhance student engagement and understanding, yet their impact on learning outcomes in oral pathology remains underexplored. Therefore, this study aimed to investigate the comparative effectiveness of role play and virtual clinical scenarios in oral pathology learning among second-year dental students. Specifically, our null hypothesis states that there is no difference in learning outcomes between virtual clinical scenario and role play.

This study is significant as it provides empirical evidence that can guide dental educators in selecting the most effective teaching tools, potentially leading to improved student performance and learning outcomes in oral pathology.

## Materials and methods

### Study design

A quasi-experimental study was conducted at Department of Oral Pathology, Dr. Ishrat-ul-Ebad Khan Institute of Oral Health Sciences, Dow University of Health Sciences (DUHS). The study was approved by Institutional Review Board of DUHS (Ref: IRB-3214/DUHS/Approval/2023/422, dated 31.10.2023) and adhered to principles of Declaration of Helsinki. The recruitment period for this study began on November 1, 2023, and ended on December 14, 2023. The study enrolled second-year undergraduate dental students who had attended all relevant lectures and tutorials and expressed willingness to participate. Exclusion criteria included students who missed any session, pre-test, post-test, as well as those with prior exposure or experience with role play and virtual clinical scenario in oral pathology or any other subjects. Withdrawal criteria allowed students to opt out of the study at any time, even after providing informed consent. Pre-test and post-test Objective Structured Practical Examinations (OSPE) were conducted before and after intervention, aligning with our institute/university exam pattern. Prior to commencing the study, students received a briefing on the study design and were assured of the confidentiality of their names and scores. They were also assured that their scores and feedback obtained from the study would not impact their formative or summative assessment. Written informed consent was obtained from these students.

### Sample size

Open epi software version 3.01 was used to calculate sample size. Based on two sample proportion with 95% confidence interval, 80% power, using mean differences reported in a previous study [19], for role play (20.63 ± 2.290) and virtual clinical scenario (21.96 ± 2.053), the calculated sample size was 84 (n = 42 per group). However, the sample size was increased to 50 students per group considering the potential dropout rates and ensure robustness in data analysis. Subsequently, 100 students were enrolled. Based on midterm scores (total score = 50), students were categorized into three groups: above average (scores ≥ 40), average (scores between 25–39), and below average (scores < 25). Within each category, students were sorted in ascending order based on their assigned random numbers. The students were then randomly divided into two groups: virtual clinical scenario group (n = 50) and role play group (n = 50). This approach minimized potential bias and ensured that the groups were comparable at baseline.

### Selection of oral lesions/conditions

A total of six cases including nicotine stomatitis, geographic tongue, aphthous ulcer, hairy tongue, oral candidiasis, and lichen planus were selected. These cases were chosen to cover a diverse range of common oral pathologies. The same cases were utilized for both the role play group and the virtual clinical scenario group to avoid measurement bias. Scripts, including patients’ demographics and case-histories, were then prepared for each scenario based on the selected cases. These topics had been previously taught in class through lectures and tutorials, ensuring that students had prior information and a basic understanding of them. The selection of topics was aligned with learning outcomes and curriculum of oral pathology at DUHS.

### Virtual clinical scenario group

In virtual clinical scenario group, clinicopathologic pictures, including clinical and histopathologic images of selected oral lesions/conditions were obtained from internet sources. Subsequently, virtual clinical scenarios were developed using Google Forms, describing dentist-patient interactions, including demographics, presenting complaint and history of presenting complaint. These scenarios were enriched by incorporating relevant images including clinical and laboratory investigation such as histopathologic images and/or other relevant laboratory report details, into the Google Forms. Finally, the link to these scenarios was shared with students in classroom. Students were instructed to read the conversation between dentist and patient, observe clinical and histopathologic images of the oral lesion, and then provide a diagnosis. The correct diagnoses were then provided in classroom. The session was conducted for four weeks. The virtual clinical scenario is detailed in Table 1.

**Table 1:** Description of role play and virtual clinical scenario.

Virtual clinical scenario	Role play
In virtual clinical scenario, a dentist-patient interaction was presented to students for examination and diagnosis. The objective was to analyze the conversation between the dentist and the patient, observe the lesion described, and provide a diagnosis using Google Forms. **Scenario overview:** Robert Johnson, a 56-year-old male patient, arrives at his dentist’s office for a routine check-up. **Activity steps:** Students were instructed to: • Examine the conversation between Dr. Anderson and Robert Johnson. • Observe the changes described by Robert Johnson in the roof of his mouth. • Use Google Forms to provide a diagnosis based on the information gathered. **Example interaction:** **Dr. Anderson:** "Good morning, Robert. I’m Dr. Anderson, and I’ll be conducting your check-up today. How have you been since your last visit?" **Robert:** "Good morning, Dr. Anderson. I’ve been okay, but I’ve noticed some changes in the roof of my mouth. It’s been bothering me for a while." **Dr. Anderson:** "I see. Have you been using any tobacco products?" **Robert:** "Yes, I have been smoking for over 30 years." **Dr. Anderson:** "Alright. Do you have any other medical conditions or take any medications regularly?" **Robert:** "Yes, I have a history of high blood pressure, and I take antihypertensive medication regularly." **Dr. Anderson:** "Thank you for letting me know. Let’s take a look at the roof of your mouth to see what might be causing these changes." Clinical and histopathologic images of nicotine stomatitis were shown.	In role play activity, teachers assumed the role of patients while students enacted as dentists. Student then recorded their diagnosis in Google Forms.**Activity steps:** • **Role assignment:** Teachers were assigned roles as patients, while students were designated as dentists. • **History taking:** The student (dentist) began by asking for demographics and inquiring about the history of the presenting complaint. • **Clinical and histopathologic presentation:** Upon completing the history taking, the teacher (patient) presented a clinical image of geographic tongue. • **Investigation:** Student then asked for investigation such as histopathological image and/or other laboratory report. The teacher then provided relevant histopathologic image/laboratory report to student. • **Diagnosis formulation:** Based on the information gathered, the student (dentist) formulated a diagnosis, discussed it with the teacher (patient).

### Role play group

As depicted in Table 1, in the role play group, the students were assigned the role of dentists while teachers acted as patients. Teachers were provided with scripts related to oral lesions. Students interacted with teachers and conducted a brief history-taking session. Once the history-taking was completed, students requested a clinical examination, teachers presented clinical images. The students then requested further investigation, and teachers showed histopathologic photographs of oral lesion to students. At the end of the activity, students presented the case history, visual parameters that aided in diagnosis and provisional diagnosis to teachers. The correct diagnoses were communicated to students by teachers. The session was conducted for four weeks.

### Learning assessment

The impact of virtual clinical scenario and role play teaching was assessed using the OSPE format, consistent with the blueprint and the DUHS exam pattern. An oral pathologist developed the OSPE stations, which featured patient history, clinical images, and histopathologic images. These stations were then reviewed and validated by two dental educationists with expertise in oral pathology and dental education. The stations were piloted on six dental students not participating in the study. To ensure consistency in difficulty levels of OSPE stations before and after intervention, item analysis, including difficulty and discrimination indices, as well as expert review by two oral pathologists were conducted. Students were instructed to record their diagnoses using a Google Form, with each correct diagnosis was assigned a score of one.

### Students’ feedback questionnaire

The feedback questionnaire regarding usefulness of virtual clinical scenario and role play on diagnostic skills, impact on learning and visual parameters were recorded using Google forms. The responses were recorded on a five- point Likert scale (strongly agree, agree, neutral, disagree and strongly disagree).

### Statistical analyses

Data were entered and analyzed using Statistical Package for the Social Sciences (SPSS) version 27.0. Shapiro-Wilk test was performed to check normality of data. Responses of questionnaire were tabulated as frequencies. The comparison between pre-test and post-test scores was conducted using the Wilcoxon signed-rank test. Mann-Whitney U test was used to compare the scores and feedback responses between the virtual clinical scenario and role play groups. For all tests, a P-value of < 0.05 was set as statistically significant.

## Results

A total of 100 students enrolled in second-year participated and no students dropped out or were absent during the study. Of them, there were 90% females and 10% males. The mean age of students was 20.21 ± 0.84 years.

Our study data did not follow normal distribution (P < 0.05); hence, non-parametric tests were considered for further analysis. The comparison between pre-test and post-test scores of virtual clinical scenario and role play groups is presented in Table 2. There was a statistically significant difference (P < 0.001) between the pre-test and post-test scores of students exposed to the virtual clinical scenario and role play (Table 2).

**Table 2:** Comparison between pre-test and post-test scores of virtual clinical scenario and role play groups.

Scores	Virtual clinical scenario group (n = 50)			Role play group (n = 50)		
	Median	Measures of dispersion (P25—P75)	P-value	Median	Measures of dispersion (P25—P75)	P-value
Pre-test	1	1–2	< 0.001*	1.50	1–2	< 0.001*
Post-test	3	2–3		3	2–3	

*P<0.05. Wilcoxon signed-rank test

The comparison of scores between virtual clinical scenario and role play is represented in Table 3. A statistically significant difference (P = 0.040) was observed in the post-test between the virtual clinical scenario and role play groups.

**Table 3:** Comparison of virtual clinical scenario and role play groups.

Group	Pre-test (n = 50)			Post-test (n = 50)		
	Median	Dispersion (P25—P75)	P-value	Median	Dispersion (P25—P75)	P-value
Virtual clinical scenario	1	1–2	0.842	1.50	1–2	0.040*
Role play	3	2–3		3	2–3	

*P <0.05. Mann-Whitney U test

An online questionnaire was used to assess students’ perception regarding usefulness of virtual clinical scenario and role play activity. Tables 4 and 5 show frequency distribution of students’ responses after role play and virtual clinical scenario session respectively.

**Table 4:** Students’ perception in role play group.

Question: I think role play	Strongly agree (%)	Agree (%)	Neutral (%)	Disagree (%)	Strongly disagree (%)	Total agreement (%)
**Diagnostic skills**						
1. helped in diagnosis	52	48	0	0	0	100
2. helped to interpret variations in clinical presentation of oral lesions	38	62	0	0	0	100
3. increased my confidence in diagnosing oral lesions without additional help	42	56	0	2	0	98
**Visual parameters**						
1. I found color of lesion important for diagnosis.	44	56	0	0	0	100
2. I found size of lesion important for diagnosis	26	54	10	10	0	80
3. I found surface appearance of lesion important for diagnosis	42	54	4	0	0	96
**Impact on learning**						
1. increased my willingness to learn oral pathology	42	54	4	0	0	96
2. made me more concentrated in learning diagnostic skills in oral pathology	42	54	4	0	0	96
3. improves in retention of knowledge related to diagnosing oral lesions	46	50	4	0	0	96
4. contribute to a more comprehensive understanding of oral pathology concepts	42	56	0	0	2	98
5. is applicable to content covered in oral pathology curriculum	44	52	2	2	0	96
6. scenarios were realistic and relevant	32	68	0	0	0	100
7. will have significant impact in my future performance	42	58	0	0	0	100

**Table 5:** Students’ perception in virtual clinical scenario group.

Question: I think virtual clinical scenario	Strongly agree (%)	Agree (%)	Neutral (%)	Disagree (%)	Strongly disagree (%)	Total agreement (%)
**Diagnostic skills**						
1. helped in diagnosis	30	64	6	0	0	94
2. helped to interpret variations in clinical presentation of oral lesions/conditions	16	76	8	0	0	92
3. increased my confidence in diagnosing oral lesions without additional help	12	56	24	8		68
**Visual parameters**						
1. I found color of lesion important for diagnosis.	18.0	72.0	8.0	0	2.0	90
2. I found size of lesion important for diagnosis	14	52	26	8	0	66
3. I found surface appearance of lesion important for diagnosis	40	56	4	0	0	96
**Impact on learning**						
1. increased my willingness to learn oral pathology	22	62	14	2		84
2. made me more concentrated in learning diagnostic skills in oral pathology	22	64	14	0	0	86
3. improves in retention of knowledge related to diagnosing oral lesions	16	70	10	4	0	86
4. contribute to a more comprehensive understanding of oral pathology concepts	18	60	20	2	0	78
5. is applicable to content covered in oral pathology curriculum	38	56	4	2	0	94
6. scenarios were realistic and relevant	14	66	20	0	0	80
7. will have significant impact in my future performance	26	56	16	2	0	82

In role play group, all students (100%) agreed that this method was beneficial in diagnosis and interpretation, with 98% expressing increased confidence. This suggests that role play is an effective tool across various aspects of diagnostic skills, underscoring its adaptability in fostering both skills and confidence levels. In virtual clinical scenario, 94% and 92% of students agreed that virtual clinical scenarios improved their diagnostic skills and assisted in the interpretation of clinical presentation of oral lesions. However, it is noteworthy that only 68% of students agreed that virtual clinical scenarios increased their confidence in diagnosing oral lesions.

Regarding visual parameters, 96% of students in both the role play and virtual clinical scenario agreed on the importance of surface appearance for diagnosing oral lesions. In role play activity, all students (100%) agreed on the importance of color, while 80% agreed on the importance of size of the lesions. In the virtual clinical scenario, 90% agreed that color is important, while only 66% agreed that size of the lesion is important for diagnosis.

In response to impact on learning, majority of students (96%) agreed that role play modality increased their willingness and concentration to learn oral pathology. Increased retention of knowledge, understanding of oral pathology concepts, applicability to the oral pathology curriculum, realistic/relevant scenarios and a greater impact in future performance were also highly agreed upon (ranging from 96% to 100%) in the context of role play. In virtual clinical scenario, while a majority agreed on positive impacts, the percentages were generally lower compared to role play, ranging from 78% to 94%. Overall, these findings suggest that role play has a more pronounced positive influence on various aspects of diagnostic skills and learning outcomes compared to virtual clinical scenario in the context of oral pathology education.

In Table 6, a comparison of students’ feedback on role play and virtual clinical scenario is presented. Feedback concerning role play perception was significantly higher than that for the virtual clinical scenario across multiple aspects (P < 0.05). Students in role play group consistently appraised role play for its usefulness in diagnosis, interpreting clinical variations, boosting confidence and addressing visual parameters and learning experiences.

**Table 6:** Comparison of students’ feedback after virtual clinical scenario and role play.

Question: I think virtual clinical scenario/role play	Group	P- value
**Diagnostic skills**		
1. helped in diagnosis	Virtual clinical scenario	0.014*
	Role play	
2. interpret variations in clinical presentation of oral lesions/conditions	Virtual clinical scenario	0.004*
	Role play	
3. increased my confidence in diagnosing oral lesions without additional help	Virtual clinical scenario	<0.001*
	Role play	
**Visual parameters**		
1. I found color of lesion important for diagnosis.	Virtual clinical scenario	0.001*
	Role play	
2. I found size of lesion important for diagnosis	Virtual clinical scenario	0.087
	Role play	
3. I found surface appearance important for diagnosis	Virtual clinical scenario	0.850
	Role play	
**Impact on learning**		
1. increased my willingness to learn oral pathology	Virtual clinical scenario	0.010*
	Role play	
2. made me more concentrated in learning diagnostic skills in oral pathology	Virtual clinical scenario	0.014*
	Role play	
3. improves in retention of knowledge related to diagnosing oral lesions	Virtual clinical scenario	<0.001*
	Role play	
4. contribute to a more comprehensive understanding of oral pathology concepts	Virtual clinical scenario	<0.001*
	Role play	
5. is applicable to content covered in oral pathology curriculum	Virtual clinical scenario	0.180
	Role play	
6. scenarios were realistic and relevant	Virtual clinical scenario	0.001*
	Role play	
7. will have significant impact in my future performance	Virtual clinical scenario	0.010*
	Role play	

*P < 0.05. Mann-Whitney U test.

## Discussion

We evaluated the effectiveness of virtual clinical scenario and role play in learning oral pathology among undergraduate dental students. Role play is defined as a learning activity in which participants are assigned specific roles to attain desired experiences. It offers the opportunity for students to harness their diagnostic and communication skills in a realistic environment [16]. Regarding students’ test scores, our study found that students in the role play session had significantly higher post-test scores compared to those in the virtual clinical scenario. This finding is in line with Al-Sebaei et al. who observed improved scores after role play session, focusing on medical emergencies in dental settings [20]. In contrast to our study, Tee et al. found significantly higher scores among students in the virtual clinics compared to those in the role play group for oral medicine/oral pathology education [19]. This difference could be attributed to variations in the study population and the degree of flexibility in utilizing virtual media. In our study, second year undergraduate dental students were exclusively exposed to either virtual clinical scenario or role play sessions within classroom settings. Conversely, in a previous study, third-year undergraduate dental students participated in role play sessions within the classroom and students in virtual media group were allowed to access materials outside the classroom at their discretion and convenience [19]. Pedrosa et al. showed significantly greater scores among trainees in virtual reality compared to role play in basic life support (BLS) training [21]. This difference is likely due to the self-training strategy in virtual reality compared to traditional role playing simulation among health professionals in BLS training. However previous studies also demonstrated that both virtual and traditional education are equally effective [22–24]. For example, Menon et al. found no significant difference in the scores between students who received training sessions on replacement of anterior teeth digitally or in a classroom setting [25]. The use of a similar set of questions in both the pre-test and post-test may account for this observation. However, our study utilized different sets of questions in the pre-test and post-test. Nevertheless, it’s worth mentioning that the questions were similar in both the virtual clinical scenario and role-play. Similarly, Sapkaroski et al. did not find any significant difference in scores between students using virtual reality and role play in radiographic positioning training [24]. Our study differs from this study in that it involves first-year students enrolled in a radiography and imaging program and utilizes student perception scores as the assessment method [11].

In our study, both role play and virtual clinical scenario groups demonstrated significantly higher post-test scores compared to their respective pre-test scores. Importantly, role play was perceived to be significantly beneficial in diagnosing and interpreting oral lesions, as well as in boosting confidence. These findings are consistent with those of Tee et al., who observed significant differences in diagnosis, interpretation and confidence between role play and virtual groups, as indicated by their post-test scores and feedback [19]. Similarly, another study reported that role play elicits a positive attitude among students and contributes to enhancing concentration, confidence, communication skills and reducing anxiety [26].

Visual parameters in tele-dentistry have gained significant attention since COVID‐19 pandemic [27]. Consequently, we asked students to identify visual parameters that aided in diagnosing oral lesions. We incorporated clinical images of white, red, and pigmented lesions in which color, size and surface appearance of the lesion reflect alterations in normal oral mucosa and thus are considered essential for diagnosis. In our study, we found that color was a crucial parameter in diagnosing oral lesions, with the size and surface appearance of lesions being equally important. In contrast, Czerninski et al., reported a different perspective, wherein surface appearance was considered as the most important parameter, while size was regarded as least important. They also noted that irregularity of the lesion emerged as the most crucial factor in diagnosis [28]. This discrepancy may be attributed to differences in case selection. For example, we utilized clinical images of nicotine stomatitis, geographic tongue, aphthous ulcer, hairy tongue, oral candidiasis, and lichen planus. In contrast, the previous study focused on clinical images of high-risk lesions such as oral squamous cell carcinoma [28].

In terms of students’ perception of the impact on learning, our study showed that role play significantly influenced various aspects of learning, including willingness, concentration, knowledge retention, and comprehensive understanding of oral pathology concepts as well as relevance to real clinical environments and potential improvements in future performance. Our findings are in accordance with previous study that highlights enthusiasm and active participation as essential elements of role play [15, 16, 19]. However, Wu et al. demonstrated that students generally preferred digital learning resources over role play due to the lack of direct communication which is known to reduce anxiety [29].

In our study, over 93% of students perceived both virtual clinical scenario and role play as equally suitable to the content covered in oral pathology curriculum. We utilized Google Forms for preparing clinical scenarios, unlike a previous study that introduced institutionally designed case-based learning resources [19], therefore it is premature to determine which method should be incorporated in curriculum. However, attempts to develop in-house digital platforms/applications and incorporate images simulating dentist-patient interactions into the curriculum should be subject to further investigation.

In summary, our study rejected the null hypothesis and demonstrated a significant difference between role play and virtual clinical scenario. Role play revealed better performance in both diagnostic skills and learning impact, highlighting the significance of experiential learning in oral pathology education. Based on our findings, we recommend that dental educationists reform teaching approaches by integrating role play into the oral pathology curriculum. Additionally, they should develop detailed scenarios, provide instructor training, and encourage student reflection to maximize the benefits of role play in oral pathology education.

The study has certain limitations which need to be mentioned. Our study findings may not be generalizable to other students, as the study was conducted in a single institute. In our four-year BDS program, oral pathology, a non-clinical subject, is taught in the second year. This differs from other Pakistani dental colleges, where this subject is typically introduced in the third year, often alongside clinical subjects such as oral medicine and periodontology. Similarly, in some other countries, oral pathology is integrated with subjects like oral diagnosis/medicine, periodontology, and oral surgery, accompanied by clinical rotations. These variations in curriculum highlight the importance of exercising caution when generalizing our findings, given the diverse program structures across institutions. Furthermore, the absence of a control group for comparison, limits our ability to determine the relative effectiveness of each intervention. Additionally, the assessment being a one-time measurement after the intervention may not fully capture the sustained impact of role play and virtual scenarios on learning outcomes. Confounding factors such as individual differences in learning styles, motivation, engagement, or interests were not assessed, potentially influencing the results obtained.

## Conclusion

Our study provides valuable insights into the usefulness of two teaching modalities, virtual clinical scenario and role play, in oral pathology instruction. Our findings suggest that role play emerges as the preferred method, significantly enhancing dental students’ learning experiences. Role play provides an interactive and immersive learning environment, facilitating the practical application of theoretical knowledge and comprehensive understanding of oral pathology concepts. Future research should investigate the long-term retention of knowledge and its practical application in clinical settings. Additionally, the impact of engagement, interest, and motivation on learning outcomes should be evaluated. It is also essential to examine the effectiveness of role-play methods for students with varying levels of motivation, interest, and engagement. Furthermore, comparing the effectiveness of role-play and virtual clinical scenarios with other teaching methods and investigating their effectiveness for different student populations will help determine their utility and applicability. These investigations will ultimately enhance oral pathology education by clarifying the benefits and potential applications of role-play and virtual clinical scenario.

## Supporting information

S1 Table Dataset of participants. (XLSX)

## References

[1] Wang W, Bi X, Zhu Y, Li X. Reforming teaching methods by integrating dental theory with clinical practice for dental students. PeerJ. 2020;8:e8477. doi: 10.7717/peerj.8477 32071811 PMC7008813

[2] Oetter N, Möst T, Weber M, Buchbender M, Rohde M, Foerster Y, et al. COVID-19 pandemic and its impact on dental education: digitalization—progress or regress? example of an online hands-on course. BMC Med Educ. 2022;22:591. doi: 10.1186/s12909-022-03638-7 35915461 PMC9340732

[3] Maatuk AM, Elberkawi EK, Aljawarneh S, Rashaideh H, Alharbi H. The COVID-19 pandemic and e-learning: challenges and opportunities from the perspective of students and instructors. J Comput High Educ. 2022;34:21–38. doi: 10.1007/s12528-021-09274-2 33967563 PMC8091987

[4] Mukhtar K, Javed K, Arooj M, Sethi A. Advantages, limitations and recommendations for online learning during COVID-19 pandemic era. Pak J Med Sci. 2020;36:27–31. doi: 10.12669/pjms.36.COVID19-S4.2785 32582310 PMC7306967

[5] Monterubbianesi R, Tosco V, Vitiello F, Orilisi G, Fraccastoro F, Putignano A, et al. Augmented, virtual and mixed reality in dentistry: a narrative review on the existing platforms and future challenges. Appl Sci. 2022;12:877. doi: 10.3390/app12020877

[6] Flores APDC, Lazaro SA, Molina-Bastos CG, Guattini VLO, Umpierre RN, Gonçalves MR, et al. Teledentistry in the diagnosis of oral lesions: a systematic review of the literature. J Am Med Inform Assoc. 2020;27:1166–72. doi: 10.1093/jamia/ocaa069 32568392 PMC7647318

[7] Perez A, Green J, Moharrami M, Gianoni-Capenakas S, Kebbe M, Ganatra S, et al. Active learning in undergraduate classroom dental education- a scoping review. PLoS One. 2023;18:e0293206. doi: 10.1371/journal.pone.0293206 37883431 PMC10602256

[8] Saluja P, Khurana C, Dave A, Arora M, Kumar S. Perception and willingness toward oral pathology and histology as a subject and profession among Indian dental undergraduates. Dent Res J (Isfahan). 2020;17:472–79. 33889354 PMC8045522

[9] Kumar PM, Gottumukkala SNVS, Ramesh KSV, Bharath TS, Penmetsa GS, Kumar CN. Effect of e-learning methods on dental education: an observational study. J Educ Health Promot. 2020;9:235. doi: 10.4103/jehp.jehp_209_20 33209927 PMC7652085

[10] Moazami F, Bahrampour E, Azar MR, Jahedi F, Moattari M. Comparing two methods of education (virtual versus traditional) on learning of Iranian dental students: a post-test only design study. BMC Med Educ. 2014;14:45. doi: 10.1186/1472-6920-14-45 24597923 PMC3975717

[11] Soltanimehr E, Bahrampour E, Imani MM, Rahimi F, Almasi B, Moattari M. Effect of virtual versus traditional education on theoretical knowledge and reporting skills of dental students in radiographic interpretation of bony lesions of the jaw. BMC Med Educ. 2019;19:233. doi: 10.1186/s12909-019-1649-0 31238927 PMC6593487

[12] Hakami Z. Comparison between virtual and traditional learning methods for orthodontic knowledge and skills in dental students: a quasi-experimental study. Healthcare (Basel). 2021;9:1092. doi: 10.3390/healthcare9091092 34574866 PMC8470017

[13] Gatt G, Attard NJ. Multimodal teaching methods for students in dentistry: a replacement for traditional teaching or a valuable addition? a three-year prospective cohort study. BMC Med Educ. 2023;23:401. doi: 10.1186/s12909-023-04377-z 37268949 PMC10236720

[14] Saigal S, Bhargava A, Kumar T, Tomar G, Kaur R, Mehta R. Evaluation of challenges encountered by dental students in the study of oral pathology: a cross-sectional study. Pak Heart J. 2023;56:25–30.

[15] McAlpin E, Levine M, Plass JL. Comparing two whole task patient simulations for two different dental education topics. Learn Instr. 2023;83:101690. doi: 10.1016/j.learninstruc.2022.101690

[16] Ganji KK, Nagarajappa AK, Sghaireen MG, Siravastava KC, Alam MK, Nashwan S, et al. Quantitative evaluation of dental students’ perceptions of the roleplay-video teaching modality in clinical courses of dentistry: a pilot study. Healthcare (Basel). 2023;11:735. doi: 10.3390/healthcare11050735 36900740 PMC10000414

[17] Ali MF, Askary G, Sajjad F. Students’ perception of the blended learning method for teaching oral pathology. Pak J Med Dent. 2022;11:88–93. doi: 10.36283/PJMD11-4/014

[18] Ali MF, Butt SA, Basim M. Perception of dental students about pbl method for constructive learning. Pak J Med Dent. 2019;8:91–6.

[19] Tee WX, Tan SH, Marican F, Sidhu P, Math SY, Gopinath D. Comparison of digital interactive case-based educational resource with virtual role play in dental undergraduates in clinical oral medicine/oral pathology education. Healthcare (Basel). 2022;10:1767. doi: 10.3390/healthcare10091767 36141379 PMC9498877

[20] Al-Sebaei MO. Evaluating the use of role-play simulations in teaching management of medical emergencies in the dental clinic. BMC Med Educ. 2023;23:831. doi: 10.1186/s12909-023-04818-9 37924046 PMC10625235

[21] Figols Pedrosa M, Barra Perez A, Vidal-Alaball J, Miro-Catalina Q, Forcada Arcarons A. Use of virtual reality compared to the role-playing methodology in basic life support training: a two-arm pilot community-based randomised trial. BMC Med Educ. 2023;23:50. doi: 10.1186/s12909-023-04029-2 36690993 PMC9869298

[22] Bains M, Reynolds PA, McDonald F, Sherriff M. Effectiveness and acceptability of face-to-face, blended and e-learning: a randomised trial of orthodontic undergraduates. Eur J Dent Educ. 2011;15:110–7. doi: 10.1111/j.1600-0579.2010.00651.x 21492347

[23] Omar H, Khan S, Toh C. Structured student-generated videos for first-year students at a dental school in Malaysia. J Dent Educ. 2013;77:640–7. doi: 10.1002/j.0022-0337.2013.77.5.tb05514.x 23658411

[24] Sapkaroski D, Mundy M, Dimmock MR. Virtual reality versus conventional clinical role-play for radiographic positioning training: a students’ perception study. Radiography (Lond). 2020;26:57–62. doi: 10.1016/j.radi.2019.08.001 31902456

[25] Menon RK, Seow LL. Development of an online asynchronous clinical learning resource ("ask the expert") in dental education to promote personalized learning. Healthcare (Basel). 2021;9:1420. doi: 10.3390/healthcare9111420 34828467 PMC8624543

[26] Mazor KM, Zanetti ML, Alper EJ, Hatem D, Barrett SV, Meterko V, et al. Assessing professionalism in the context of an objective structured clinical examination: an in-depth study of the rating process. Med Educ. 2007;41:331–40. doi: 10.1111/j.1365-2929.2006.02692.x 17430277

[27] Flores-Hidalgo A, Collie J, King S, Grant FT, Beasley NE, Moss ME, et al. The use of teledentistry in clinical oral and maxillofacial pathology practice: an institutional experience. Front Oral Health. 2023;4:1063973. doi: 10.3389/froh.2023.1063973 37546293 PMC10398386

[28] Czerninski R, Mordekovich N, Basile J. Factors important in the correct evaluation of oral high-risk lesions during the telehealth era. J Oral Pathol Med. 2022;51:747–54. doi: 10.1111/jop.13343 36053963 PMC9544116

[29] Wu YJA, Lan YJ, Huang SBP, Lin YTR. Enhancing medical students’ communicative skills in a 3d virtual world. J Educ Techno Soc. 2019;22:18–32.

